# Association of decreased variation of coefficient R–R interval with ischemic colitis and small bowel obstruction

**DOI:** 10.1371/journal.pone.0228117

**Published:** 2020-02-12

**Authors:** Toshio Arai, Hiroki Yamada, Takeya Edagawa, Satoshi Yoshida, Shigetoshi Hikimoto, Hiromichi Sougawa, Kenichiro Nakachi

**Affiliations:** 1 Department of Gastroenterology, Hashimoto Municipal Hospital, Hashimoto, Wakayama, Japan; 2 Department of Cardiology, Hashimoto Municipal Hospital, Hashimoto, Wakayama, Japan; University Magna Graecia of Catanzaro, ITALY

## Abstract

**Background:**

The parasympathetic nervous system exerts and controls intestinal tone. Several studies have suggested that the coefficient of the R–R intervals (CVRR) is useful for evaluating the parasympathetic nervous system.

**Objectives:**

This study aimed to evaluate the relationship between gastrointestinal emergencies, specifically ischemic colitis (IC) and small bowel obstruction (SBO), and the autonomic nervous system.

**Methods:**

In this retrospective study, a total of 13 patients with IC or SBO aged ≧65 years were analyzed. CVRR was measured in patients with IC and SBO and controls.

**Results:**

CVRR averaged to 8.8% ± 2.5% in controls, 1.4% ± 0.4% in patients with IC, and 2.4% ± 1.0% in SBO groups (p < 0.001). CVRR was significantly lower in patients with IC and SBO than that in controls.

**Conclusion:**

The results of this study demonstrate the possibility that CVRR may serve as a clinical index for assessing the functioning of the parasympathetic nervous system in patients with IC or SBO.

## Introduction

Gut mobility is controlled by the autonomic nervous system. Gastrointestinal diseases such as irritable bowel syndrome (IBS) and paralytic bowel obstruction (BO) are caused by autonomic nervous dysfunction [1–3]. In these disease states, medications to stimulate the autonomic function are commonly prescribed [4]. The autonomic function of IBS has been investigated via the coefficient of variance of R–R interval (CVRR), which has been reported to be significantly lower in patients with IBS than in healthy controls [1]. CVRR is a variable that is derived from electrocardiography, which commonly used to evaluate the parasympathetic nerve system. CVRR is non-invasive and widely available and is thus used for the follow-up of the autonomic function of patients with diabetes and Parkinson’s disease [5–9]. Kudoh et al. reported that a decrease in CVRR is caused by the anticholinergic effects of chronic neuroleptics and that this parameter is useful for the evaluation of postoperative paralytic ileus in patients with chronic schizophrenic [10]. However, CVRR is only used for specific gastrointestinal diseases.

Gastrointestinal emergencies such as ischemic colitis (IC) and BO are commonly accompanied by abdominal pain and constipation. These symptoms are commonly considered to be caused by reduced gut motility [11–13].

IC generally occurs in the large intestine and is caused by the diminished perfusion of the colon, which leads to mucosal injury [11, 12, 14]. Colonic blood flow may be compromised by a change in the systemic circulation or by anatomic functional changes in the local mesenteric vasculature. This is more likely to occur in individuals aged ≧65 years with cardiac arrhythmia or thrombophilia [12]. A patient’s clinical history, stool culture, computed tomography (CT) scans, and colonoscopy findings are essential for diagnosis [15, 16]. However, colonoscopy is associated with a high risk of complications in elderly individuals [17]. In certain clinical situations such as gangrenous IC, a colonoscopy is contraindicated owing to the risk of perforation [16]. For these reasons, IC diagnosis needs to be based on other modalities such as CT.

BO is another gastrointestinal emergency, with a 75% incidence in the small intestine; in such cases, it is known as small intestinal obstruction (SBO). BO is divided into the following two types: (1) mechanical, which is caused by adhesion after surgeries and (2) non-mechanical [18]. The application of a colonoscopy is limited based on the location of BO, especially in SBO. Accordingly, CT is commonly used for diagnosis [3, 13, 18, 19].

However, for both IC and SBO, several concerns are associated with CT such as radiation exposure and contrast administration. Certain conditions such as a history of allergies, asthma, renal disease, and myeloma increase the risk of adverse reactions owing to the administration of contrast agents [20]. Therefore, a different clinical diagnostic tool is required. CVRR is a promising method to assess the parasympathetic nervous system in patients with IC and SBO. This study aimed to evaluate whether CVRR is feasible to assess the autonomic nerve activation in patients with IC and SBO.

## Methods

This retrospective study was approved by the Ethical Committee of Hashimoto Municipal Hospital. All subjects were adults and were provided with written information about the study prior to obtaining consent. Verbal informed consent was obtained by doctors and documented in the list. From June 1, 2018, to December 31, 2018, patients with clinical and radiographic evidence of IC and SBO who visited our hospital were registered in this study. We excluded cases of arrhythmia and patients treated for diabetes mellitus, Parkinson’s disease, schizophrenia, IBS, or functional dyspepsia. Also, there were no patients taking medication that affecting the autonomic nervous function. In all patients, CVRR was measured for 3 min using an ECG machine (EDG-9522; Fukuda Denshi, Tokyo, Japan). We used the Fisher’s exact test to assess male to female ratio between the groups. The Kruskal–Wallis test was used to compare age as well as CVRR between groups. If the Kruskal–Wallis test indicated a significant difference, the Steel–Dwass test was used. All analyses were performed using the GraphPad Prism version 5.01 (GraphPad Software, San Diego, CA). A *P*-value of <0.05 was considered to be significant.

## Results

**Fig 1:**
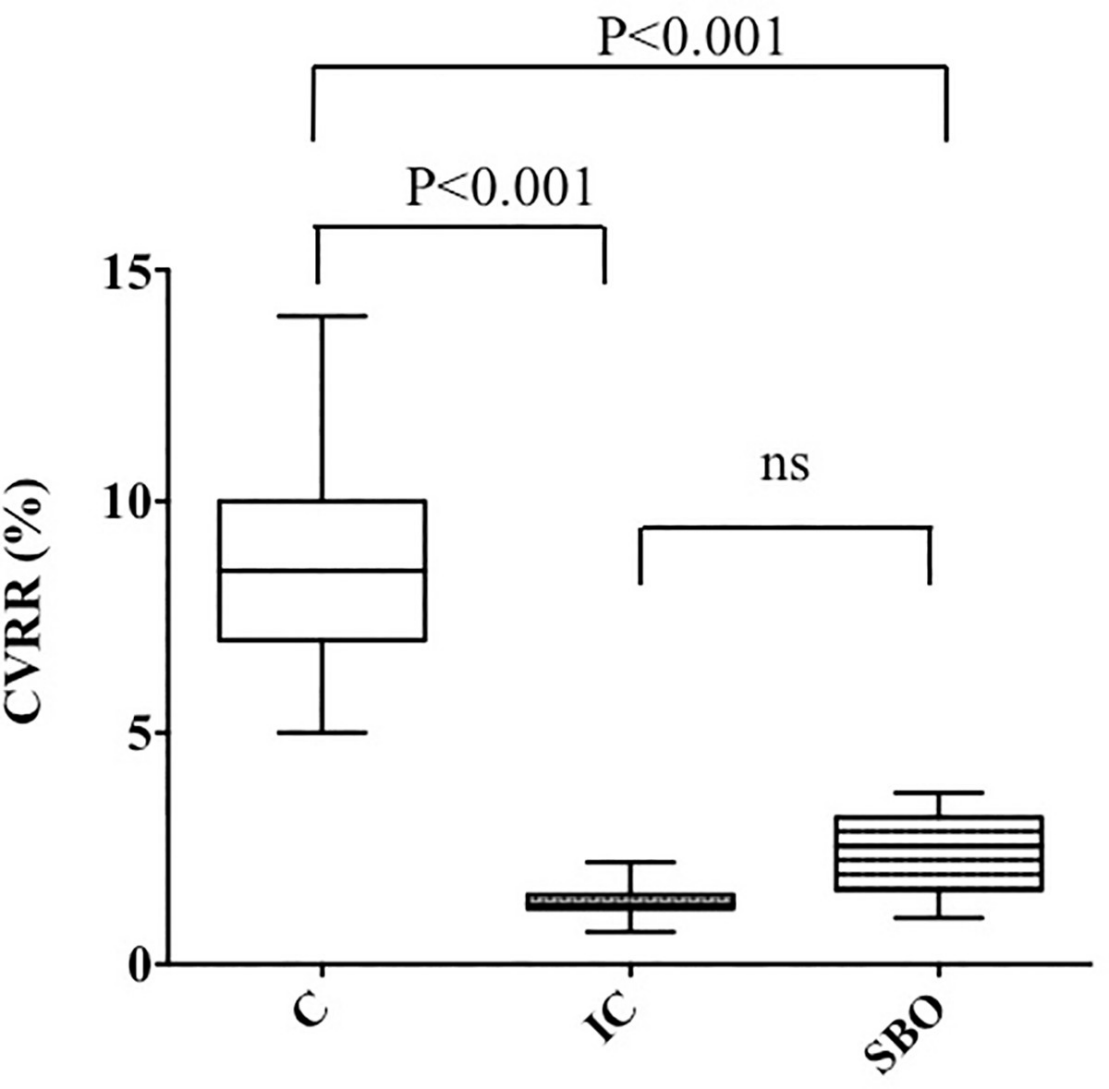
CVRR on the electrocardiogram. All values are expressed as means ± SEM; *P*-value is determined as using Kruskal–Wallis test with Steel–Dwass test. C = Control, IC = Ischemic Colitis, SBO = Small Bowel Obstruction, ns = not significant.

A total of 13 patients with IC or SBO aged ≧65 years were analyzed. The present study comprised seven patients with IC (six females and one male), six patients with SBO (four females and two males), and 14 control patients (10 females and 4 males). The clinical data of 27 patients are shown in Table 1. There was no statistically significant difference in the mean age or male-to-female ratio between the groups. As shown in Fig 1. CVRR averaged to 8.8% ± 2.5% in controls, 1.4% ± 0.4% in patients with IC, and 2.4% ± 1.0% in the SBO groups (p < 0.001), whereas there was no significant difference between the IC and SBO group.

**Table 1:** Clinical characteristics of subjects.

	Control	IC	SBO	*P*
N	14	7	6	
Female, n (%)	10 (71.4)	6(85.7)	4 (66.7)	0.62
Age(Mean ± SD)	77.5 ± 9.0	79.0 ± 9.3	81.3 ± 10.1	0.57

Data presented as frequency (%) of patients.

All values are expressed as means ± SD. There is not a significant difference between groups. C = Control, IC = Ischemic Colitis, SBO = Small Bowel Obstruction.

## Discussion

Our results demonstrated a significant decrease in CVRR in patients with IC and SBO, suggesting that parasympathetic nerve activation may be correlated in these patients.

Our study showed the feasibility of the clinical application of CVRR in patients with gastrointestinal conditions. As CVRR is a non-invasive, longitudinal observation using this tool is possible. CVRR may allow an early detection and follow-up tool postoperative SBO. In addition, if applied preoperatively, CVRR may work as a predictive index for the occurrence of postoperative SBO, however, to distinguish between IC and postoperative SBO, CT may be necessary as additional information. CVRR is affected by various factors such as age and comorbidities. To eliminate such bias, we set the inclusion criteria as age ≧ 65 years and no comorbidities including arrhythmia, diabetes mellitus, Parkinson’s disease, schizophrenia, IBS, and functional dyspepsia. Considering that the normal range of CVRR differs between age groups [8], the diagnostic threshold of CVRR may change according to age. Moreover, if the patient is taking medication that affects autonomic nervous function, baseline CVRR may be modified and the age-dependent threshold cannot be applied. Even in such cases, the longitudinal application of CVRR may be considered.

This study has several limitations. First, data were collected in a retrospective manner analyzed with a small sample size. A large number of longitudinal observations of CVRR with the clinical course of IC and SBO should be considered in future studies. Second, comparisons with another marker such as sympathetic activity were not performed. A more detailed ECG-based method such as determining the ratio of low-frequency to high-frequency fluctuation in heart rate [2, 7] could be considered.

In conclusion, CVRR is a feasible index to assess autonomic dysfunction in the clinical assessment of patients with IC and SBO.

## Supporting information

S1 Table Patient characteristics for 27 patients. (DOCX)
