# Supplementary material for: Association of decreased variation of coefficient R–R interval with ischemic colitis and small bowel obstruction
Source: PLoS One. 2020 Feb 12;15(2):e0228117. doi: 10.1371/journal.pone.0228117 (PMC7015401; doi:10.1371/journal.pone.0228117)
Supplement: S1 Table — (DOCX) [file pone.0228117.s001.docx]

S1 Table  Patient characteristics for 27 patientss.

| Patient No | Group | Age (years) | Sex | CVRR |
| --- | --- | --- | --- | --- |
| 1 | C | 66 | M | 5.0 |
| 2 | C | 82 | M | 9.0 |
| 3 | C | 72 | F | 7.0 |
| 4 | C | 65 | F | 8.0 |
| 5 | C | 92 | F | 10.0 |
| 6 | C | 82 | F | 8.0 |
| 7 | C | 86 | F | 6.0 |
| 8 | C | 71 | F | 9.0 |
| 9 | C | 84 | F | 7.0 |
| 10 | C | 85 | M | 14.0 |
| 11 | C | 65 | F | 9.0 |
| 12 | C | 70 | F | 8.0 |
| 13 | C | 81 | F | 10.0 |
| 14 | C | 84 | M | 13.0 |
| 15 | IC | 78 | F | 1.5 |
| 16 | IC | 76 | F | 1.3 |
| 17 | IC | 66 | M | 2.2 |
| 18 | IC | 91 | F | 1.5 |
| 19 | IC | 91 | F | 0.7 |
| 20 | IC | 72 | F | 1.3 |
| 21 | IC | 79 | F | 1.2 |
| 22 | SBO | 93 | F | 3.7 |
| 23 | SBO | 85 | M | 2.8 |
| 24 | SBO | 84 | F | 1.0 |
| 25 | SBO | 71 | F | 2.3 |
| 26 | SBO | 67 | F | 1.8 |
| 27 | SBO | 88 | M | 3.0 |

Group: C = Control, IC = Ischemic Colitis, SBO = Small Bowel Obstruction.

Gender: F = Female, M = Male,
